# Supplementary material for: α2-Adrenergic modulation of Ih in adult-born granule cells in the olfactory bulb
Source: Front Cell Neurosci. 2023 Jan 6;16:1055569. doi: 10.3389/fncel.2022.1055569 (PMC9853206; doi:10.3389/fncel.2022.1055569)
Supplement: Supplementary file 1 [file Data_Sheet_1.pdf]

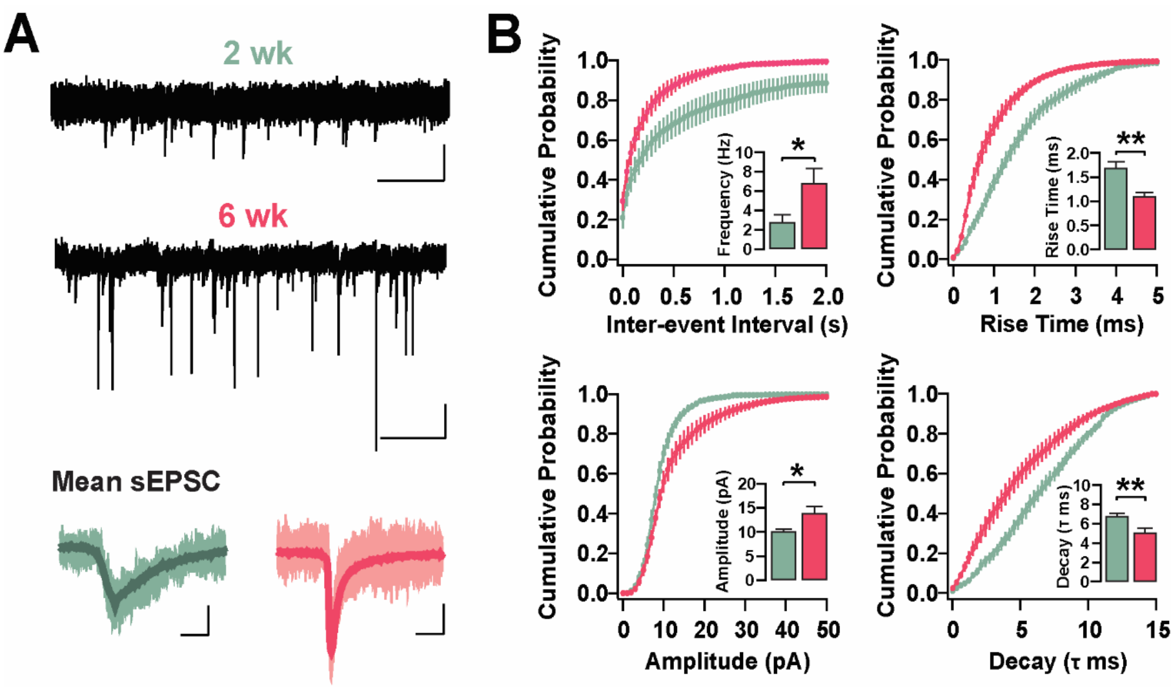

Supplementary Figure 1. Changes in the kinetic properties of excitatory synapses in adult-born GCs

(A) Top: representative voltage-clamp recordings at −70 mV showing spontaneous excitatory post-synaptic currents (sEPSCs) in GCs at 2 wk and 6 wk post-labeling (pink, 2 wk: n = 12 cells; green, 6 wk: n = 13 cells). The calibration is 10 pA and 500 ms. Bottom: average sEPSC waveforms from GCs at 2 and 6 wk, illustrating changes in amplitude, rise time, and decay with age. The calibration is 5 pA and 5 ms. **(B)** Average cumulative probability distributions for inter-event intervals, amplitude, rise time and decay for the sEPSCs in GCs at 2 and 6 wk post-labeling. Insets show the average frequencies and amplitudes (p = 0.02), and rise times and decays (p = 0.003) of sEPSCs at these two time points post-labeling.

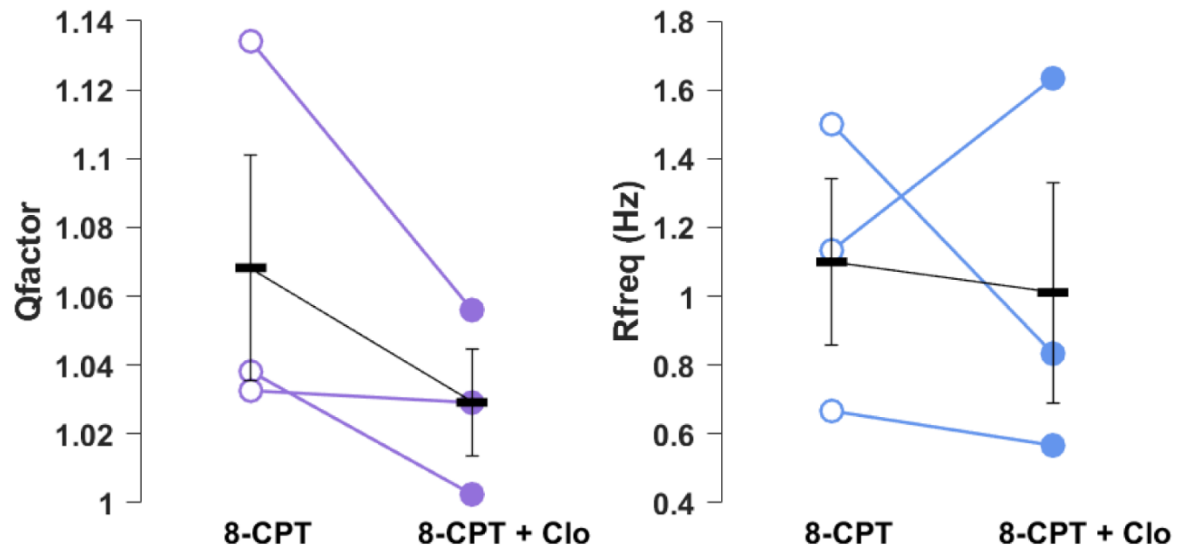

Supplemental Figure 2. cAMP prevents decrease in resonance upon α2-AR activation. Left**,** there is no significant reduction in the Q-factor upon α2-AR activation with pre-application of 8-CPT-cAMP. Rigth, there is no significant reduction in resonant frequency upon α2-AR activation with of 8-CPT-cAMP.

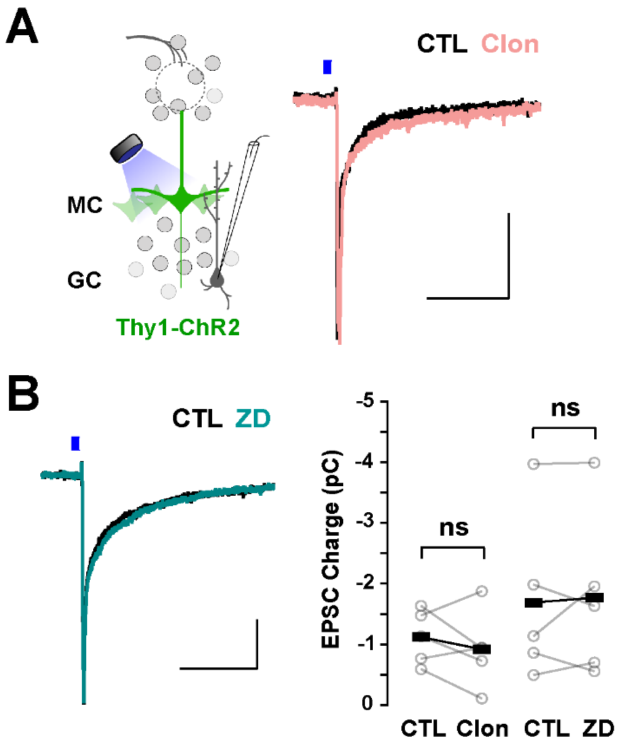

Supplementary Figure 3. Blockade of I_h and activation of α2-ARs does not affect the MC to GC synapses

(A) Left, diagram of the strategy used to elicit lateral inhibition. We recorded from GCs while optogenetically activating the MCs in OB slices from Thy1-ChR2 mice. Right, sample traces of light-elicited EPSCs in GCs at –70 mV during control (black) and in the presence of Clon (pink). The calibration bar is 10 pA and 200 ms. **(B)** Left, sample traces of light-elicited EPSCs in GCs during control (black) and in the presence of ZD. Right, summary plot of EPSC charge transfer showing no significant change in under both experimental conditions (Clon, n = 5 cells, p = 0.39; ZD, n = 5 cells, p = 0.73).
